# Optimization-Based Capacitor Balancing Method with Selective DC Current Ripple Reduction for CHB Converters

**DOI:** 10.3390/en15010243

**Published:** 2021-12-30

**Authors:** Luis Galván, Pablo Jesús Gómez, Eduardo Galván, Juan Manuel Carrasco

**Affiliations:** Electronical Engineering Department, University of Seville, 41092 Seville, Spain; pgomez@gte.esi.us.es (P.J.G.); egalvan@us.es (E.G.)

**Keywords:** capacitor balance, cascaded H-bridge converter (CHB), common-mode voltage, current ripple, multilevel converter, optimal control, pulse-width modulation (PWM)

## Abstract

From its introduction to the present day, Cascaded H-Bridge multilevel converters were employed on numerous applications. However, their floating capacitor, while advantageous for some applications (such as photovoltaic) requires the usage of balancing methods by design. Over the years, several such methods were proposed and polished. Some of these methods use optimization techniques or inject a zero-sequence voltage to take advantage of the converter redundancies. This paper describes an optimization-based capacitor balancing method with additional features. It can drive each module DC-Link to a different voltage for independent maximum power point tracking in photovoltaic applications. Moreover, the user can specify the independent active power set points to modules connected to batteries or any other energy storage systems. Finally, DC current ripple can be reduced on some modules, which can extend the lifespan of any connected ultra-capacitors. The method as a whole is tested on real hardware and compared with the state-of-the-art. In its simplest configuration, the presented method shows greater speed, robustness, and current wave quality than the state-of-the-art alternative in spite of producing about 1/3 fewer commutations. Its other characteristics provide additional functionalities and improve the adaptability of the converter to other applications.

## 1. Introduction

**Figure 1:**
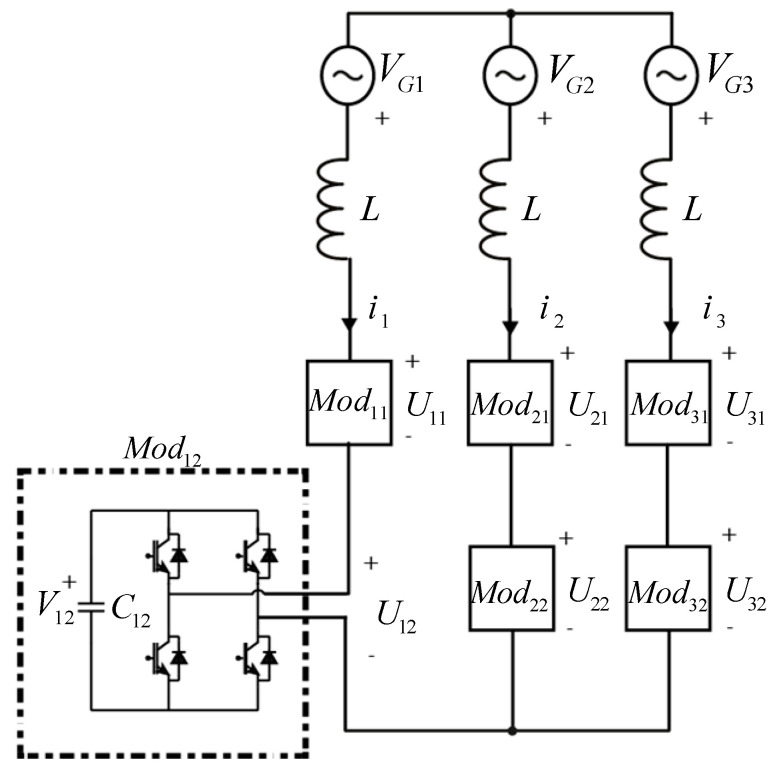
Scheme of the CHB topology. The figure also shows the nomenclature considered in this paper, including the sign criteria and the ordering of the subscripts.

Cascaded H-Bridge (CHB) converters have supposed a big step forward in the development of Multilevel converters technology. This type of modular converter is increasingly widespread in the industry, due to the great number of advantages compared with the traditional converters [1]. From its introduction, this topology, shown in Figure 1, is mainly used in photovoltaic plants [2,3,4,5,6,7,8], Static Synchronous Compensators (STATCOM) [9,10,11,12,13], and power distribution applications [14,15,16].

Related investigations focus on enhancing the capability and efficiency of this converter [17,18]. This can be achieved through improvements in control strategies and in the voltage balance method. In some applications, such as photovoltaic generation or STATCOM or when combining energy storage systems (ESS) of different technologies [19], it is preferable to have independent voltage set points, meaning some imbalance between both phases and modules [20]. Several methods have already been presented to equalize the DC-Link of the modules.

Some of these balancing strategies are usually applied as part of the modulation. First, the current regulator calculates the voltage reference for the converter, and then, the corresponding balancing method adjusts this voltage reference independently for some or all modules. This way, each module can be commanded to absorb or deliver a certain amount of power in order to regulate their DC-Link voltage ([21,22] show some examples). New trends in this field are introducing a new concept of modulation, modifying either the carrier shape (using trapezoidal PWM [23,24]) or the way in which modulation is implemented [25,26]. These methods have the disadvantage of distorting the current exchanged with the grid. They are also complicated to apply when the number of modules is high.

Strategies in other publications have modified their control algorithm to add the balancing objective. In [2,27], triple voltage harmonics are added to balance the modules. Following this trend, a zero-sequence voltage can be included to guarantee the balance between the phase modules, as disclosed in [28,29]. However, these methods can only have a limited effect on the unbalance of the modules because they depend on the usage of the DC-Link voltages.

New investigations are treating the balancing problem as part of a global optimization problem. In this case, the common-mode redundancy is exploited by evaluating all the possible states of the converter. In [30], a Finite Control Set Model Predictive Control (FCS-MPC) was developed to ensure the voltage balance between modules. However, the high number of modules output combinations presents an important computational cost when attempting to apply this strategy to CHB converters. The absence of a fixed switching frequency is another disadvantage. For those reasons, some new investigations, such as [9], focus on reducing the number of iterations of the MPC.

Finally, a new method was presented in [31] to control all the DC-Link voltages independently without interfering with the grid current. It has later been expanded in [32] to further consider a power set point for modules comprising ultra-capacitors or other ESSs. This method considers all freedom degrees and calculates the optimal output of each module without having to iterate through all the possibilities, thus reducing the computational cost. Although the method performed well in the simulations, no experimental results were presented in those publications.

The present paper further elaborates on this last method, unifying its description and presenting several novelties. First of all, the method is now tested on a real 20 kVA CHB converter instead of simply through simulations, therefore validating it beyond the theory. The implementation of the method on real control hardware is disclosed, justifying its capability to respond in real-time in spite of having to solve an optimization problem on each control cycle.

Secondly, the method is now compared with newer and more robust state-of-the-art techniques than it was in [31], where the alternative method was shown not to support step references. The comparison made in the present paper further includes the impact of the methods on the current wave and the number of commutations. These were only vaguely considered in previous publications, and no comparison was conducted with any other method.

Finally, the present paper explores more deeply the effect of tuning and configuring the method. This was not performed in [31], where all modules were considered to be equal and only superficially considered in [32], where modules difference was very limited. In the present paper, several tests were run where modules were treated differently, showing the effect of different settings combinations. This justifies the method advantages on different applications, especially on hybrid converters with different devices connected to the modules DC-Links.

The rest of this paper is structured as follows. Section 2 describes the fundaments and mathematical approach of the proposed method. Section 3 describes the materials and methods employed for the tests. It also includes a brief description of the steps followed by the proposed method as well as the state-of-the-art method it is compared with. Section 4 describes the tests and shows their results, which are later discussed in more detail in Section 5. The paper conclusions are also included in Section 5.

## 2. Fundaments and Strategy

A typical control scheme for a CHB comprises two main layers. The current regulation layer achieves the desired exchange of active and reactive power by regulating the phase currents. The output of this regulation is a reference for the total voltage to be modulated by each phase: $U_{T1}{}^{*}$, $U_{T2}{}^{*}$, and $U_{T3}{}^{*}$. Afterward, the modulation layer selects one of the many possible ways to produce these voltages by means of pulse width modulation (PWM). This selection usually aims to balance the voltages of the DC-Link modules using the many converter redundancies. Since only phase-to-phase voltages need to be observed, the modulation layer may add any common-mode voltage to $U_{T1}{}^{*}$, $U_{T2}{}^{*}$ and $U_{T3}{}^{*}$, as performed in [28,29].

The method proposed in this paper is designed for this modulation layer. In addition, to balance the DC-Links, or regulate their voltage independently, it intends to lower the voltage and current ripple by penalizing the power deviation. These objectives are weighed by their corresponding user-defined gains, which may be configured independently for each module. Thus, on a CHB with 3 phases and 2 modules per phase, there are 12 configurable gains in total: one related to the voltage and one related to the power on each of the 6 modules. These gains will be introduced as $G_{Vkj}$ and $G_{Pkj}$, respectively, in the following subsections.

### 2.1. DC-Link Independent Voltage Control

The first objective is to minimize the deviation of each DC-Link voltage from a certain voltage set point. A possible function to measure this deviation is

(1) $$F_{V}=\overset{3}{\underset{k=1}{\sum }}\overset{N}{\underset{j=1}{\sum }}\frac{1}{2}G_{Vkj}C_{kj}{\left(V_{kj}{}^{*}-V_{kj}\right)}^{2}$$

where $V_{kj}$ and $V_{kj}{}^{*}$ are the actual and desired DC-Link voltages of module kj. The function also includes the module DC-Link capacity $C_{kj}$ thus that it has units of energy (Joules) and a dimensionless gain $G_{Vkj}$ to weigh each module as desired.

It is worth noting that $F_{V}$ does not depend directly on any control signal. Therefore, the proposed method aims to minimize its time-derivative value. This way, $F_{V}$ will be led to its minimum as fast as possible. This is similar to a Lyapunov approach, where the control action is selected according to the time-derivative of the Lyapunov function. Here, instead of making this derivative always negative, it is minimized. Thus, if there is any way for $F_{V}$ to decrease, it will do so as quickly as possible; otherwise, $F_{V}$ will increase as slowly as possible. The time-derivative of $F_{V}$ is

(2) $$\frac{dF_{V}}{dt}=\overset{3}{\underset{k=1}{\sum }}\overset{N}{\underset{j=1}{\sum }}G_{Vkj}\left(V_{kj}{}^{*}-V_{kj}\right)i_{k}\cdot \frac{-U_{kj}}{V_{kj}}$$

where $i_{k}$ is the current in phase k and $U_{kj}$ is the modulated output voltage of module kj. Minimizing this time-derivative is equivalent to maximizing an objective function $f_{V}$, which is linear with the modules output voltages.

(3) $$f_{V}=\overset{3}{\underset{k=1}{\sum }}\overset{N}{\underset{j=1}{\sum }}B_{Vkj}U_{kj}$$

(4) $$B_{Vkj}=\frac{G_{Vkj}i_{k}\left(V_{kj}{}^{*}-V_{kj}\right)}{V_{kj}}$$

$B_{Vkj}$ represents the benefit of increasing the output voltage of module kj by 1 volt. Thus, if only $f_{V}$ was intended to be minimized, $U_{T1}{}^{*}$, $U_{T2}{}^{*}$, and $U_{T3}{}^{*}$ would be allocated thus that modules with high $B_{Vkj}$ modulate the highest possible voltage and modules with low $B_{Vkj}$ modulate the lowest possible voltage.

### 2.2. Active Power Control and Ripple Reduction

As a second objective, the proposed method aims for each module to receive active power $P_{kj}$ to follow a certain set point $P_{kj}{}^{*}$. Depending on the DC-Link, this may have different advantages. On the one hand, on modules with batteries or other ESSs, the DC-Link voltage $V_{kj}$ will not change much as power flows into or out of the module. Thus, controlling the module power $P_{kj}$ will produce more accurate results than attempting to control the module voltage $V_{kj}$. On the other hand, on modules whose DC-Link only consists of capacitors $P_{kj}{}^{*}$ can be set to zero to minimize the ripple. Ultra-capacitors benefit from both advantages: they can be given a power set point when they must be charged or discharged and a zero power set point when their current ripple is meant to be minimal.

The instantaneous power received by the module is expected to have a ripple at twice the grid frequency. Deviations from this behavior must be penalized in an objective function. This way, when the power reference is null, any power exchange, and thus any current producing a voltage ripple on the DC-Link, will be penalized. Since the modulation method can only select the module’s output voltages (not the phase currents), this power deviation is expressed as a voltage deviation weighed by the current that flows through the module.

The module output voltage corresponding to the desired power is

(5) $$U_{kj}{}^{*}=\frac{3i_{k}P_{kj}{}^{*}}{i_{\alpha }{}^{2}+i_{\beta }{}^{2}}=\frac{3i_{k}P_{kj}{}^{*}}{i_{d}{}^{2}+i_{q}{}^{2}}$$

where $i_{\alpha }$, $i_{\beta }$, $i_{d}$ and $i_{q}$ represent the *αβ* and *dq* currents according to the power-invariant transformations. The actual output voltage of the module can be parameterized as

(6) $$U_{kj}=U_{kj}{}^{*}+U_{Akj}+U_{Bkj}$$

where $U_{Akj}$ is nonnegative and $U_{Bkj}$ is nonpositive. Voltages $U_{Akj}$ and $U_{Bkj}$ represent the deviation of $U_{kj}$ from its desired value $U_{kj}{}^{*}$ in the corresponding direction (positive or negative). An objective function $f_{P}$ can be defined to measure the voltage deviation of the module output in terms of $U_{Akj}$ and $U_{Bkj}$. In order for $f_{P}$ to have the same units and structure as $f_{V}$, the absolute value deviation was selected instead of the typical quadratic deviation.

(7) $$f_{P}=\overset{3}{\underset{k=1}{\sum }}\overset{N}{\underset{j=1}{\sum }}B_{PAkj}U_{Akj}+B_{PBkj}U_{Bkj}$$

(8) $$B_{PAkj}=-G_{Pkj}\left|i_{k}\right|$$

(9) $$B_{PBkj}=G_{Pkj}\left|i_{k}\right|$$

$B_{PAkj}$ and $B_{PBkj}$ represent the marginal benefit of increasing $U_{Akj}$ and $U_{Bkj}$, similar to $B_{Vkj}$. It is worth noting that $U_{Bkj}$ is always positive and $B_{PAkj}$ is always negative, thus there is always some benefit in having $U_{kj}$ becoming closer to $U_{kj}{}^{*}$. If only $f_{P}$ was to be minimized, the output of modules with higher absolute values of $B_{PAkj}$ and $B_{PBkj}$ would be intended to follow $U_{kj}{}^{*}$ more accurately and vice versa.

The dimensionless gain $G_{Pkj}$ can be configured by the user to weigh the power deviation as desired for each module. $G_{Pkj}$ is expected to be null on modules intended for reactive power exchanges as it would otherwise interfere with this power exchange.

### 2.3. Complete Linear Optimization Problem

In order for both objectives to be considered, the global objective function f to maximize is the addition of the previous objective functions $f_{V}$ and $f_{P}$. The global benefits $B_{Akj}$ and $B_{Bkj}$ can also be obtained by adding together the benefits corresponding to $f_{V}$ and $f_{P}$.

(10) $$f=f_{V}+f_{P}=\overset{3}{\underset{k=1}{\sum }}\overset{N}{\underset{j=1}{\sum }}B_{Akj}U_{Akj}+B_{Bkj}U_{Bkj}$$

(11) $$B_{Akj}=B_{Vkj}+B_{PAkj}=\frac{G_{Vkj}i_{k}\left(V_{kj}{}^{*}-V_{kj}\right)}{V_{kj}}-G_{Pkj}\left|i_{k}\right|$$

(12) $$B_{Bkj}=B_{Vkj}+B_{PBkj}=\frac{G_{Vkj}i_{k}\left(V_{kj}{}^{*}-V_{kj}\right)}{V_{kj}}+G_{Pkj}\left|i_{k}\right|$$

It is worth noting that the objective function f is linear with $U_{Akj}$ and $U_{Bkj}$. The values of $B_{Akj}$ and $B_{Bkj}$ can be calculated from the currents and voltages measured on each control cycle. Thus, they can be considered constants for the optimization problem.

The optimization is subject to some constraints. In particular, the modules output voltages $U_{kj}$ selected by the method must be compatible with the phase-to-phase voltages selected by the current regulation layer.

(13) $$\begin{array}{c} \overset{N}{\underset{j=1}{\sum }}U_{1j}-U_{2j}=U_{T1}{}^{*}-U_{T2}{}^{*}\\ \overset{N}{\underset{j=1}{\sum }}U_{2j}-U_{3j}=U_{T2}{}^{*}-U_{T3}{}^{*} \end{array}\}$$

Since the desired output voltages $U_{kj}{}^{*}$ are known on each control cycle, they can be subtracted from the desired phase voltages $U_{Tk}{}^{*}.$ This way the constraints can be expressed in dependence of $U_{Akj}$ and $U_{Bkj}$.

(14) $$U_{Tk}{}^{\prime }=U_{Tk}{}^{*}-\overset{N}{\underset{j=1}{\sum }}U_{kj}{}^{*}$$

(15) $$\begin{array}{c} \overset{N}{\underset{j=1}{\sum }}U_{A1j}+U_{B1j}-U_{A2j}-U_{B2j}=U_{T1}{}^{\prime }-U_{T2}{}^{\prime }\\ \overset{N}{\underset{j=1}{\sum }}U_{A2j}+U_{B2j}-U_{A3j}-U_{B3j}=U_{T2}{}^{\prime }-U_{T3}{}^{\prime } \end{array}\}$$

Any output combination that meets the constraints given by (15) will produce the phase-to-phase voltages required by the current regulation layer. Among those valid combinations, the one that maximizes the global objective function f is the optimal output. Thus, the proposed method for the modulation layer consists of selecting the output corresponding to the solution of the LOP given by (16).

(16) $$\begin{array}{c} \max\overset{3}{\underset{k=1}{\sum }}\overset{N}{\underset{j=1}{\sum }}B_{Akj}U_{Akj}+B_{Bkj}U_{Bkj}\\ \overset{N}{\underset{j=1}{\sum }}U_{A1j}+U_{B1j}-U_{A2j}-U_{B2j}=U_{T1}{}^{\prime }-U_{T2}{}^{\prime }\\ \overset{N}{\underset{j=1}{\sum }}U_{A2j}+U_{B2j}-U_{A3j}-U_{B3j}=U_{T2}{}^{\prime }-U_{T3}{}^{\prime }\\ U_{Akj}\in \left[0,\;V_{kj}-U_{kj}{}^{*}\right]\\ U_{Bkj}\in \left[-V_{kj}-U_{kj}{}^{*},\;0\right] \end{array}\}$$

## 3. Materials and Methods

### 3.1. Materials

**Figure 2:**
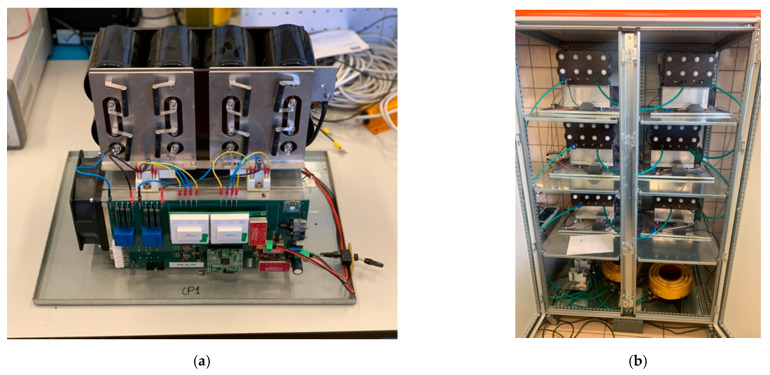
One individual module (**a**) and the whole converter (**b**) where the tests have been run.

The method was tested on a 20 kVA-power laboratory converter with 6 H-Bridge modules. Figure 2a shows one module, and Figure 2b shows the whole converter. The modules are arranged as in Figure 1, with 2 modules per phase. Table 1 shows the main characteristics of the converter.

Although the nominal power of the converter was 20 kVA, the presented results correspond to tests at lower power, where the current THD was higher. This evidences the differences in the methods performance regarding the switching, the DC-Link voltage, and the current modulation. In addition, since the converter does not include ESSs other than the DC-Link capacitors, the set points for the modules active power $P_{kj}{}^{*}$ were always set to 0. This way, even though the charge and discharge of ESSs were not tested, it was possible to test the DC current ripple reduction and its dependence on the method gains.

The converter control hardware was distributed among a central control unit and 6 local control units (one on each module). The central control unit mainly consists of a microprocessor and an FPGA, which exchange information with one another. The microprocessor was a Texas Instruments eZdsp TMS320F28335, which runs most of the algorithm. Its debug software was employed as the human-machine interface. The FPGA is a Xilinx Spartan-6 XCM-206 Series; it manages the communication with the local control units and maintains them synchronized. The central control unit is connected to all local control units through optical fiber to ensure galvanic isolation between modules. Each local control unit is managed by a Xinlinx Spartan3A model XC3S200A-4VQG100C, which is connected to various sensors and IGBT drivers.

The local control units measure their DC-Link voltages ($V_{kj}$), the phase currents ($i_{k}$), and the grid voltages ($V_{Gk}$). This information was sent to the central control unit, which executes the control algorithm to select a duty cycle for each module. Then, the central control unit sends the duty cycle back to the local control units along with a synchronization signal. This synchronization signal indicates the beginning of the triangular carrier for the pulse width modulation (PWM). The local control units convert their respective duty cycles to modulation signals and activate the IGBTs accordingly. Regarding the modulation, a symmetric triangular carrier was used with 2 executions of the control cycle for each triangular period.

Regarding the test bench, the converter was connected to a laboratory source, which emulates a 400 V and 50 Hz grid. The source was a California Instruments MX30, whose protections were configured to open the circuit if the current of any phase became greater than 20 A. A Yokogawa DLM4058 oscilloscope was employed to measure the either the DC-Links voltages ($V_{kj}$) or the modules outputs ($U_{kj}$). The switching of the latter provides information regarding the module’s commutations. The grid current harmonics and THD were measured with a Fluke 434 grid analyzer.

### 3.2. Methods

**Figure 3:**
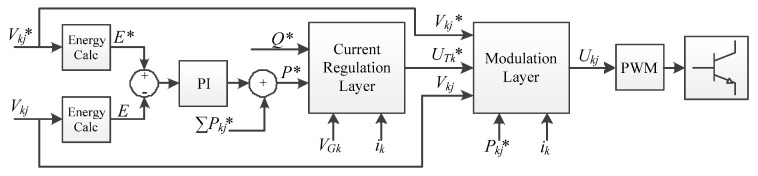
General control scheme. The proposed method as well as the state-of-the-art alternative belong in the modulation layer.

The full control method is summarized in Figure 3.

The user selects the set points for each DC-Link voltage $V_{kj}{}^{*}$, each module active power $P_{kj}{}^{*}$ and the total reactive power $Q^{*}$. As previously mentioned, the actual DC-Link voltages $V_{kj}$, the grid voltages $V_{Gk}$, and currents $i_{k}$ were measured by the local control units and transmitted to the central control unit. With this information, the microprocessor calculates the total energy E stored among all the DC-Links, as well as the desired energy $E^{*}$ that corresponds to the DC-Links voltage set points. According to these values, a PI regulator selects the active power $P^{*}$ for the converter to receive from the grid. If any module had a nonzero active power set point $P_{kj}{}^{*}$, then it would be added to the total active power $P^{*}$ as a feedforward term.

A typical current regulation algorithm was employed to meet these active $P^{*}$ and reactive $Q^{*}$ power set points. In particular, a classic dq approach was considered. The grid voltages and currents were passed to dq axes, references for the d and q currents were obtained according to $P^{*}$ and $Q^{*}$, and controlled using two PI regulators. The voltages selected by the regulators were passed back from dq axes to abc axes. The resulting voltage references $U_{Tk}{}^{*}$ were then passed to the modulation layer.

Two methods were considered for the modulation layer. The first one was the method proposed in the present paper. To evaluate the advantages of the first method, it was compared with known state-of-the-art techniques, which were employed together as a second method. Both complete methods are described below in their respective subsections. Either way, the output of the modulation layer was the selected voltages $U_{kj}$ to be modulated by the converter.

Duty cycles were obtained accordingly and sent to the local control units, which modulate the actual voltages. Although the duty cycles could be calculated on the local control units (by passing them the values of $U_{kj}$) doing so produced more DC-Link voltage ripple and worse phase current THD. Hence, it was decided to send the duty cycles to ensure a more consistent modulation.

#### 3.2.1. Proposed Method

The proposed method consists of following these steps:

1. Obtain $B_{Akj}$ and $B_{Bkj}$ as in Equations (11) and (12), respectively.
2. Obtain $U_{kj}{}^{*}$ as in Equation (5).
3. Obtain $U_{Tk}{}^{\prime }$ as in Equation (14).
4. Obtain $U_{Akj}$ and $U_{Bkj}$ by solving the LOP in Equation (16).
5. Obtain $U_{kj}$ as in Equation (6).

The fourth step requires solving a LOP with some particular properties on every control cycle. A possible algorithm to do so was presented by the same authors in [31,32]. The algorithm is summarized here, but please refer to [31,32] for more details.

First, the benefit values ($B_{Akj}$ and $B_{Bkj}$) of all modules of the same phase were sorted from greatest to smallest. It is worth noting that, for any module, $B_{Akj}$ is always smaller than (or equal to) $B_{Bkj}$ thus the set is partially sorted. For N modules per phase, the FPGA on the central control unit can solve such a list of benefits in 2N−2 clock cycles doing up to N independent comparisons per clock cycle. However, the converter used for the test was small enough (N=2) for the sorting to be performed by the microprocessor. The merge sorting algorithm was selected because for in this case it can sort each list in 3 comparisons.

After sorting, voltages $U_{Tk}{}^{\prime }$ are assigned to variables $U_{Akj}$ and $U_{Bkj}$ in the order of the to the benefit values. As a result, on each phase there was one basic variable, which was not saturated. Variables with a higher benefit than that of the basic variable were high-saturated, whereas variables with a lower benefit than that of the basic variable were low-saturated. This operation was iterative and required up to 2N−1 iterations for each phase.

Then, the 3 benefit values corresponding to the basic variables were added together. If the result was positive, then there was some benefit in increasing the common-mode voltage and vice versa. To increase the common-mode voltage, all the basic variables were increased equally until one of them became saturated. This variable was no longer basic, and the next one of the same phase (in decreasing order of benefit value) became the new basic variable of the phase. A similar procedure was applied to decrease the common-mode voltage: all basic variables were decreased until one of them was saturated, and the previous variable of the same phase replaced it as the new basic variable. Either way, the benefits of the new set of basic variables were added together again and the process repeated iteratively. The optimal solution was found when the sum of the benefit values changed sign or when all variables of the same phase were saturated on the same direction (all high or all low). In the very worst case, there can be 6N−3 iterations. This limit can be lowered if the current regulation layer selects $U_{Tk}{}^{*}$ with no common-mode voltage (which is typical).

It is worth noting that, as long as the FPGA can perform N comparisons simultaneously, all parts of the solving algorithm scale linearly with N. This is advantageous because it means that each additional module added to the converter will require about the same amount of extra time than the previous one. It also allows estimating the limit number of modules that a converter may have depending on control hardware and the acceptable response time.

#### 3.2.2. State-of-the-Art-Method

The second possible method for the modulation layer comprised 2 main steps:

1. Select the common-mode voltage.
2. Distribute each phase voltage reference among the modules of that phase.

The first step aims to manage the energy distribution among the 3 phases of the converter, whereas the second further managed this energy distribution among the modules of each phase. Several publications addressed these goals separately. Here, a representative candidate algorithm was selected for each one.

For the first step, the method from [28] was employed. This method adds a zero-sequence voltage depending on a power reference for each phase. The employed formula is

(17) $$v_{0}=-\sqrt{\frac{2}{3}}\frac{\left(\begin{array}{cc} 1 & 1 \end{array}\right)}{{\left|i_{\alpha \beta }^{*}\right|}^{2}}\left(\begin{array}{cc} 2p_{1}^{*}-p_{2}^{*}-p_{3}^{*} & 0\\ 0 & \sqrt{3}\left(p_{2}^{*}-p_{3}^{*}\right) \end{array}\right)\left(\begin{array}{c} i_{\alpha }^{*}\\ i_{\beta }^{*} \end{array}\right)$$

where $v_{0}$ is the zero-sequence voltage and $p_{1}{}^{*}$, $p_{2}{}^{*}$, and $p_{3}{}^{*}$ are the phase power references. The sign and scale are different from those in [28] because of the voltage sign criteria and the αβ transformation, which here is the power-invariant.

The power references $p_{k}{}^{*}$ are selected in proportion to the sum of DC-Link voltage deviations on each phase. For coherence, the proportional gain is the same as the one used on the PI before the current regulation layer (see Figure 3). Although adding an integral action was considered, all attempts to do so were unsuccessful due to instability.

Once $v_{0}$ is obtained, it is added to the three phases reference voltages $U_{T1}{}^{*}$, $U_{T2}{}^{*}$, and $U_{T3}{}^{*}$. The second step consisted of distributing these phase reference voltages among the modules of their corresponding phase. To do so, the balancing method from [33] was selected. This method sorts the DC-Link voltages and starts assigning output voltages starting from the highest or from the lowest depending on the current sign. Since each DC-Link could have a different voltage set point, the set point was subtracted from each DC-Link voltage before sorting.

## 4. Tests and Results

Unlike previous publications [31,32], where individual parts of the method were tested only with simulations, the present paper includes hardware tests results that finally validate the whole method. Several tests were made to check the general performance of the proposed method. In addition, the effect of tuning the method gains was explored more deeply than in previous publications. Whenever the proposed method and the aforementioned state-of-the-art alternative shared the same objective (the latter does not consider individual power set points), their results and performance were compared and contrasted. Each test is described in its own subsection.

To avoid ambiguity, each module was assigned a different color for the oscilloscope representation. These colors, indicated in Table 2, are employed when representing DC-Link voltage and commutations.

### 4.1. Permanent State

This test checks the converter steady-state behavior when employing either method. For the test, the reactive power set point $Q^{*}$ is set to 5 kVAr, and the voltage set point $V_{kj}{}^{*}$ is set to 200 V for all modules. The proposed method is configured to simply balance the DC-Links and treat all modules equally. This is conducted by selecting $G_{Vkj}=1$ and $G_{Pkj}=0$ on all modules.

Table 3 shows the phase current harmonic content for each method. Since the current regulation is equal for both methods, only the DC-Link ripple and the modulation are expected to affect the current harmonic content. The proposed method has a higher seventh harmonic, and the state-of-the-art one has a higher fifth harmonic, but they both have approximately the same THD.

**Figure 4:**
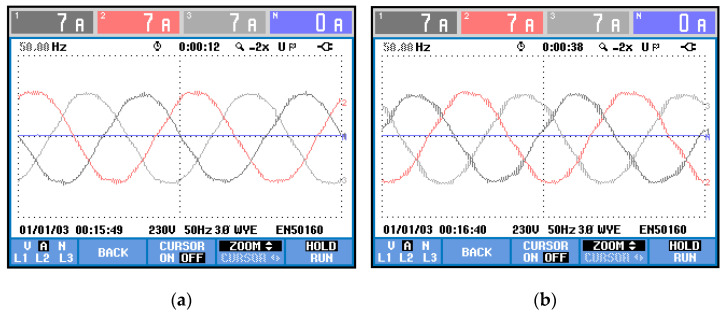
Phase currents wave shape: (**a**) proposed method; (**b**) state-of-the-art alternative.

However, the grid analyzer does not consider the higher frequency harmonics when obtaining the THD, thus it is best also to check the shape of the current waves on each case. According to Figure 4, the phase current is even cleaner from harmonic content when using the proposed method.

**Figure 5:**
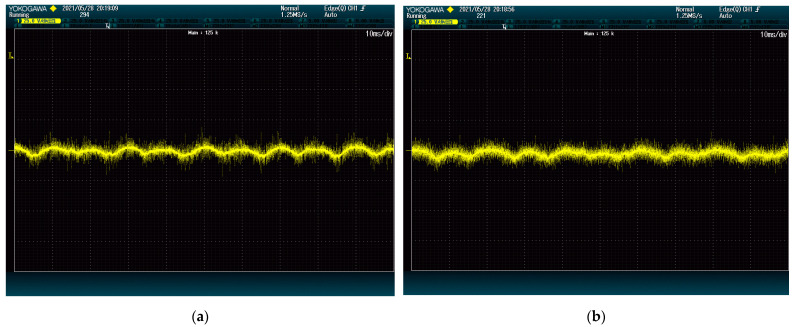
DC-Link voltage ripple: (**a**) proposed method; (**b**) state-of-the-art alternative. The plots show voltage (25 V/div) vs. time (10 ms/div).

Figure 5 shows the voltage ripple of an individual module when applying either method. There is no meaningful difference between them, aside from some noise; both methods produce about 15 V ripple. Results from the next test confirm that, for these gains, this similarity persists.

**Figure 6:**
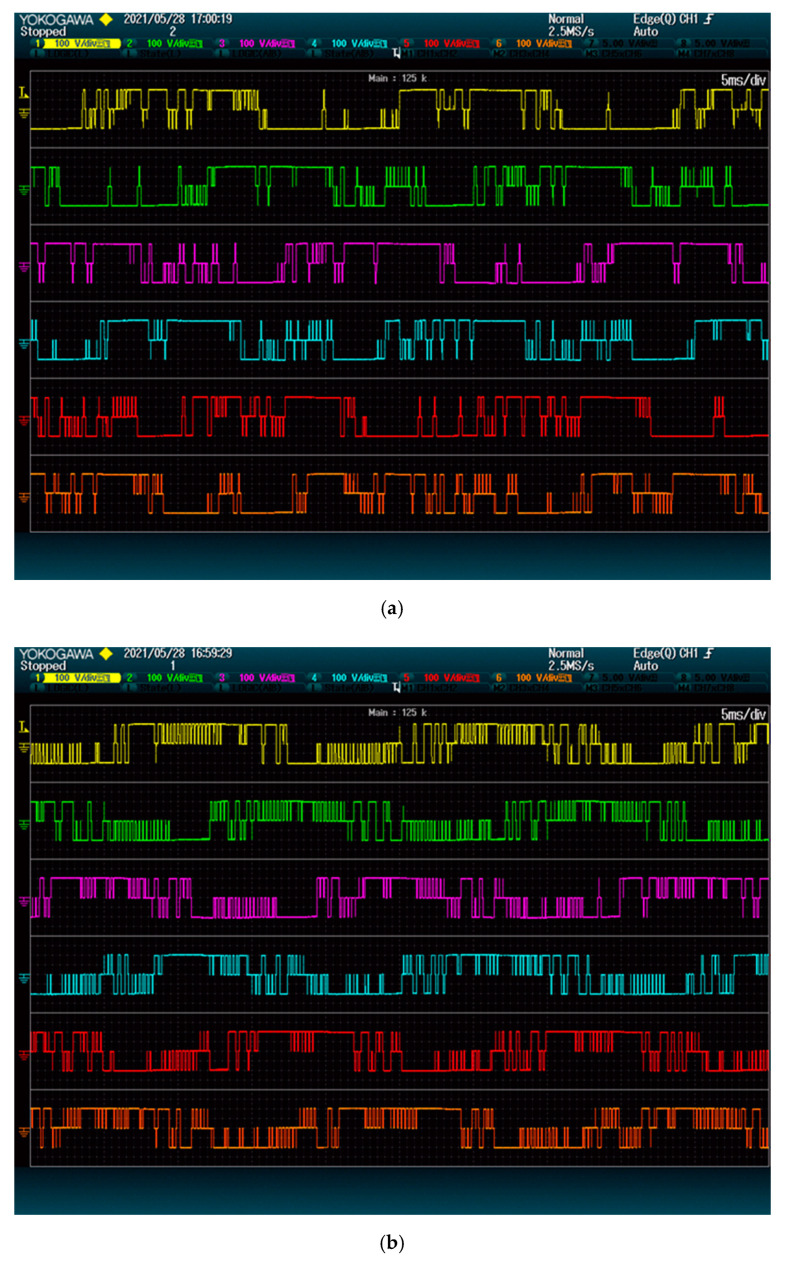
Modules output: (**a**) proposed method; (**b**) state-of-the-art alternative. The plots show 2.5 grid periods.

Figure 6 shows the modules switching for each method. The proposed method clearly has a lower number of commutations on all modules. This is so because the proposed method only considers phase-to-phase voltages when solving the LOP. Thus, it behaves more similarly to the space vector modulation, with two commutations per control cycle (instead of 3). The state-of-the-art alternative, on the other hand, considers the voltage of each phase separately, thus having three commutations per control cycle.

In general, it can be said that the proposed method can achieve the same ripple and better quality current than the state-of-the-art alternative with a reduced amount of commutations.

### 4.2. Response upon a Step Reference

This test checks the DC-Link dynamics upon step references. The steps are selected thus that the total energy in the converter does not change. Otherwise, the active power reference would change, and the dynamic would be due to the current regulation layer. Each module is given a different set point $V_{kj}{}^{*}$ ranging from 200 V to 250 V. Once the steady-state has been reached, set points are swapped with one another using a step reference. All set points are changed at once, and the result is captured by the oscilloscope. The proposed method gains remain as on the previous test: $G_{Vkj}=1$ and $G_{Pkj}=0$ on all modules.

**Figure 7:**
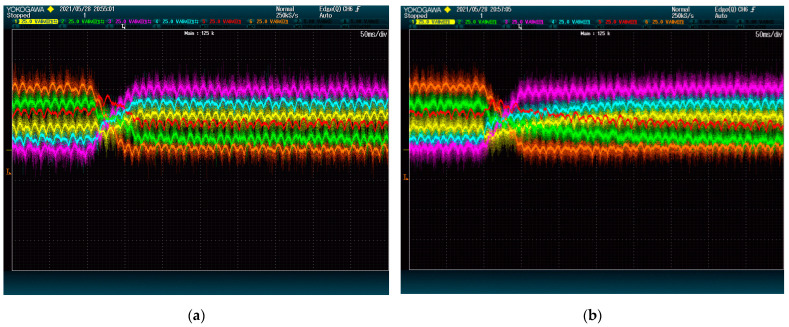
DC-Link voltage dynamics upon set point swap at 5 kVAr: (**a**) proposed method; (**b**) state-of-the-art alternative. The plots show voltage (25 V/div) vs. time (50 ms/div).

**Figure 8:**
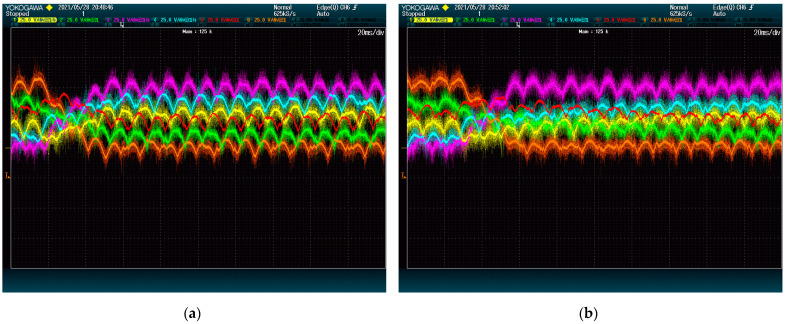
DC-Link voltage dynamics upon set point swap at 9 kVAr: (**a**) proposed method; (**b**) state-of-the-art alternative. The plots show voltage (25 V/div) vs. time (20 ms/div).

Figure 7 and Figure 8 show the result of the test at 5 kVAr and 9 kVAr, respectively. These figures show that reactive power has an important impact on the speed of the DC-Link dynamics. This is so because both methods rely on the converter current to move energy between modules.

It is worth noting that, regardless of the reactive power, the voltages of 4 DC-Links (given by red, yellow, green, and blue lines) are shown to reach their destination faster with the proposed method than with the alternative. This difference is due to the ways the methods deal with power being transferred between phases. In this case, the modules of the first phase (blue and yellow) need to receive power from the modules of the second phase (red and green). The method from [28] achieves this power exchange by using a voltage zero-sequence, which is shown to be less effective than the strategy of the proposed method. The voltage of the other two modules (orange and purple), which belong to the same phase, evolves similarly for both methods. This shows that the performance difference is exclusively due to the common-mode voltage management.

**Figure 9:**
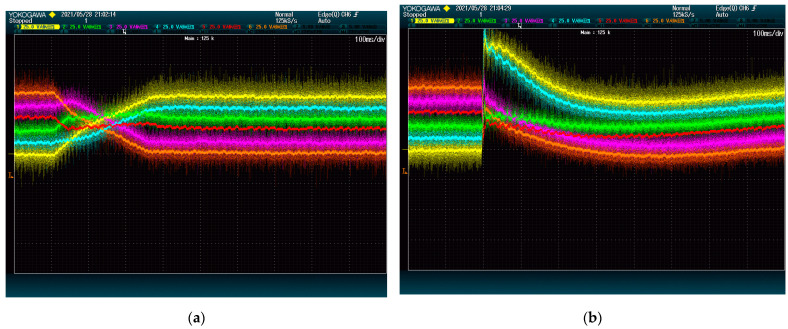
DC-Link voltage dynamics upon set point swap at 1 kVAr: (**a**) proposed method; (**b**) state-of-the-art alternative. The plots show voltage (25 V/div) vs. time (100 ms/div).

Since the performance depends so much on the reactive power, the test is run one more time at 1 kVAr. This time, the voltages are selected thus that a high amount of power must flow from the third phase to the first one. As shown in Figure 9, the proposed method still achieves its goal without incidents, whereas the method from [28] produces a high peak. This exemplifies the proposed method’s stability.

As previously mentioned, the method from [28] depends on three-phase power references ($p_{1}{}^{*}$, $p_{2}{}^{*}$ and $p_{3}{}^{*}$), which in return are obtained from a proportional regulator. Reducing the regulator gain might reduce the peak, but it would also make the method performance even slower. The proposed method, on the other hand, retains its aforementioned speed without having to be tuned for each particular situation. This evidences the robustness and adaptability of the proposed method.

### 4.3. Effect of the Voltage Gains

The proposed method can be configured to prioritize some modules over others. This is not considered by the state-of-the-art method, thus it cannot be compared. This test checks the effect of having different gain values for each module. To do so, all DC-Link voltages are given a step reference from 180 V to 250 V at the same time with different combinations of $G_{Vkj}$. The test is run at 5 kVAr and $G_{Pkj}$ remains null for all modules.

**Figure 10:**
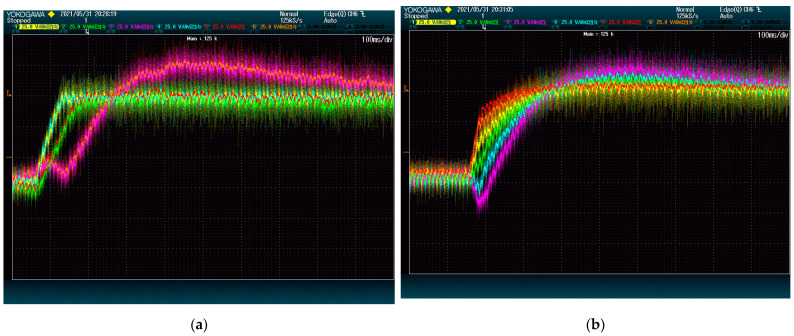
DC-Link voltage dynamics upon a reference step with different voltage gains: (**a**) gain by phase; (**b**) gain by module. The plots show voltage (25 V/div) vs. time (50 ms/div).

Figure 10 shows the results of the test. In Figure 10a, modules of the same phase have equal voltage gain: $G_{Vkj}=1$ for the first phase, $G_{Vkj}=0.1$ for the second one and $G_{Vkj}=0.01$ for the third. Although all DC-Link voltages tend asymptotically to their set point, they do so at different speeds and with a different degree of precision. Modules belonging to the first phase (yellow and blue lines) are charged first, even though the converter currents remain balanced. Modules of the second phase (green and red) reach their set point later, and modules of the third phase (purple and orange) are not paid so much attention by the algorithm.

In Figure 10b, each module is given a different gain. Sorted from highest to lowest, gain values are: $G_{V22}=10$ (red), $G_{V32}=6.8$ (orange), $G_{V11}=4.7$ (yellow), $G_{V21}=3.3$ (green), $G_{V12}=2.2$ (blue) and $G_{V31}=1.5$ (purple). Again, modules with the highest gains are charged faster, even at the cost of temporary discharging modules with lower gains.

This shows that it is possible to prioritize some voltages over others and even configure how much important each one is in relation to the others.

### 4.4. Ripple Reduction

This final test checks the method capacity to selectively reduce the DC-Link current and voltage ripple. To do so, $G_{Pkj}$ set to 0.1 for the first module of each phase (yellow, green, and purple) and zero for all the others. All modules power set points $P_{kj}{}^{*}$ are set to 0, as their DC-Link is not meant to receive power but to move low (ideally null) current. The test is performed on a steady-state at 5 kVAr with all DC-Links set points at 200 V and all voltage gains $G_{Vkj}$ are set to 1. DC-Link voltages and module switching are monitored.

**Figure 11:**
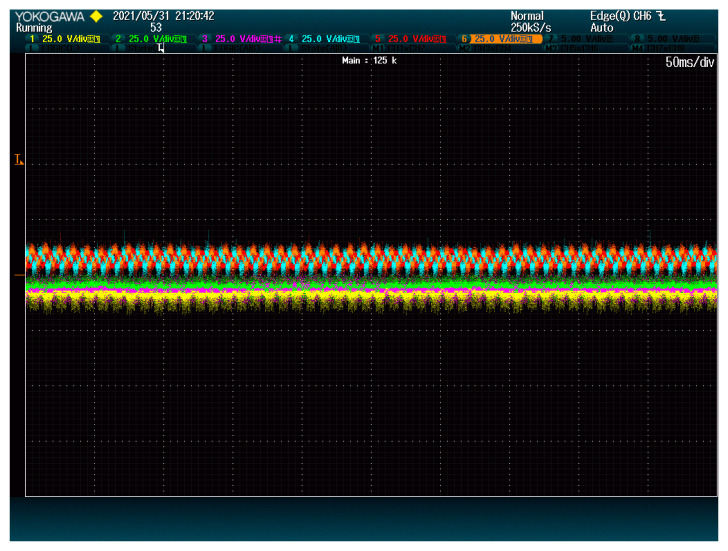
Modules DC-Link voltage when reducing ripple. The plot shows voltage (25 V/div) vs. time (50 ms/div).

As shown in Figure 11, the voltage ripple is almost nonexistent in the selected modules. The only ripple for the yellow, green, and purple lines seems to be due to the signal noise. Since these DC-Links only comprised capacitors, the voltage ripple is proportional to the current ripple, which means that the DC current ripple is also significantly reduced.

However, this reduction comes at a cost: DC-Link voltages are now slightly deviated because reducing the ripple is not totally compatible with maintaining all the voltages balanced. The proportion between $G_{\mathrm{Pkj}}$ and $G_{\mathrm{Vkj}}$ can be tuned according to this tradeoff to find the desired solution.

**Figure 12:**
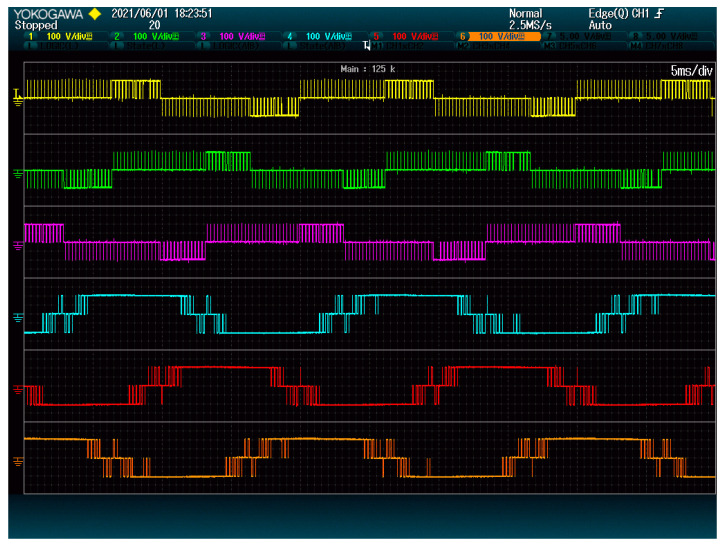
Modules switching when reducing ripple. 2.5 grid periods are shown.

Figure 12 shows that the total amount of commutations has increased, and they now are concentrated on the modules with positive values of $G_{\mathrm{Pkj}}$. This means that modules whose ripple is meant to be limited must be designed to support a higher effective switching frequency. Fortunately, this frequency is limited by the triangular carrier. In addition, the other modules now need to stand a lower number of commutations than before, as shown in the same figure.

## 5. Discussions and Conclusions

Results show that the proposed method has several advantages over the state-of-the-art. As a modulation method, it has a reduced number of commutations, similar to an αβ space vector modulation method. The effective commutation frequency is thus about 2/3 of the original. Nevertheless, the produced current has better quality than the state-of-the-art alternative. The lower performance of the aforementioned alternative may be due to the zero-sequence voltage injection, which could be producing saturations when the reactive power is low. It may be possible to prevent some of these saturations by reducing the proportional gain of the method from [28], but doing so would also reduce its speed and balance capability.

The DC-Link voltage ripple is also similar for both the proposed method and the state-of-the-art alternative. This contrasts with the simulations previously published in [31], where the proposed method was supposed to greatly reduce the voltage ripple even on its most basic configuration ($G_{Vkj}=1$ and $G_{Pkj}=0$ on all modules).

As a balancing method, it can distribute energy between modules of the same phase as well as between different phases. For modules of the same phase, the method performs just as effectively as sorting the modules by DC voltage and allocating the output voltage accordingly (as in [33]). When balancing modules between different phases, it behaves better than a zero-sequence voltage injection method (such as [28]). Thus, the proposed method combines both possibilities and produces an improved result.

Upon step references, the proposed method dynamic is faster and more robust than the zero-sequence voltage injection method. In addition, it is shown to be more adaptable, not requiring tuning any additional parameter, depending on the reactive power exchange. This is so because the proposed method is optimization-based and, consequently, searches for the best possible solution given the circumstances. The method automatically adjusts its speed depending on the converter power, thus it remains very robust and stable without having to slow down unnecessarily.

It is also justified in the present paper that the algorithm to solve the optimization problem is relatively easy to scale. The necessary time to find the optimal solution can scale linearly with the total number of modules, as long as the employed FPGA can do N comparisons at once (N being the number of modules per phase). This means that the central FPGA programmable area also needs to scale linearly with the number of modules per phase, which is consistent with the usage of the FPGA as a communication tool with all the modules.

Publications such as [30], which also consider optimization methods, propose to combine the current regulation layer and the modulation layer in only one control method. While this type of method could potentially perform better than the proposed method, they need to consider all the possible converter output combinations. This is extremely hard to scale because the number of solutions to consider increases exponentially with the number of modules on a CHB converter. The proposed method, on the other hand, supports a larger number of modules.

In addition to surpassing state-of-the-art as a balancing method, the proposed method has other important features, which make it appropriate for several applications.

1. It can consider different voltage set points for each module, which is useful for maximum power point tracking (MPPT) on photovoltaic applications.
2. It can consider power set points for some or all modules. These work better than voltage set points on modules connected to batteries, whose voltage is approximately constant with their energy.
3. It can reduce the DC current ripple on some modules. On modules with ultra-capacitors, this can extend the life of the ultra-capacitors.
4. It can consider different priorities on some modules, making it more adaptable to converters where modules have different applications.

The first, third, and fourth advantages have been tested on the converter with good results, only the third one is shown to have some drawbacks. The second advantage has not been tested yet due to the need for batteries to connect to the converter, and it is expected to be tested in the future.

These advantages allow the method to be employed on hybrid applications. For example, consider a CHB where some modules are connected to photovoltaic panels while others are not. The former can produce active power, but their voltage must be controlled for MPPT; whereas the latter is only required to increase the output voltage by providing reactive power. The priority of the former modules can be increased by assigning them a greater $G_{Vkj}$ gain value. In this way, the method will ensure that these modules have the desired DC voltage, while the others absorb any energy perturbation.

This converter may also include batteries to store some of the produced energy. By configuring $G_{Vkj}$ and $G_{Pkj}$, it is possible to give power set points to the batteries and voltage set points to the other modules.

Another application would be a STATCOM, where some modules have been connected to ultra-capacitors for some short active power transfer to the grid. When the ultra-capacitors are not needed to charge or discharge, their modules can be assigned a positive value for $G_{Pkj}$ to limit their ripple.

The usage of $G_{Pkj}$ for either power set points or ripple reduction was shown to produce additional commutations. It was also noticeable that applications that required these features were related to energy storage systems (batteries and ultra-capacitors), whose voltage was usually low. Since the corresponding modules would have to stand lower voltage and higher switching frequency, MOSFETs seem to be more appropriate. The usage of the proposed method may lead to converters combining MOSFETs modules with IGBT modules, obtaining the best of each transistor technology.

In conclusion, the proposed and tested method is shown to be faster, more robust and easier to tune, and more scalable than the state-of-the-art balancing methods. It also presents special features that make it especially appropriate for different applications, even opening the way for hybrid applications. In fact, because of its general design, it can be seen as a general modulation method for almost any CHB converter.

## Figures and Tables

**Table 1:** Converter characteristics.

Parameter	Value	Parameter	Value
Nominal phase-to-phase voltage	400 V	Type of transistors	IGBT
Nominal RMS phase current	30 A	Maximum DC-Link voltage	800 V
Phase inductance (L)	6 mH	DC-Link capacitance (C_kj_) ^1^	4.1 mF
Control frequency	4 kHz	Modulation carrier frequency	2 kHz

^1^ All DC-Links have the same capacitance.

**Table 2:** Oscilloscope color for each module.

Module	Phase 1	Phase 2	Phase 3
First	Yellow	Green	Purple
Second	Blue	Red	Orange

**Table 3:** Current harmonic content.

Method	Phase	Harmonic Amplitude (%)										THD (%)
		2nd	3rd	4th	5th	6th	7th	8th	9th	10th	11th	
Proposed method	1st	1.0	1.1	0.8	2.7	0.4	1.4	0.3	0.2	0.2	0.5	3.6
	2nd	1.1	1.0	0.9	2.1	0.4	1.6	0.3	0.2	0.2	0.5	3.4
	3rd	0.9	1.2	0.8	2.7	0.3	1.5	0.2	0.2	0.2	0.4	3.6
State-of-the-art alternative	1st	1.0	1.0	0.8	3.0	0.4	0.9	0.3	0.2	0.2	0.3	3.6
	2nd	1.1	1.0	0.7	2.8	0.4	1.1	0.3	0.2	0.2	0.4	3.5
	3rd	0.8	0.9	0.7	2.8	0.4	1.0	0.2	0.2	0.2	0.2	3.4

## Nomenclature

**Variables Notation**, **Meaning**, **and Introduction**		
**Symbol**	**Meaning**	**Definition**
$B_{Akj}$	Global benefit of increasing $U_{Akj}$ towards the total objective function	(11)
$B_{Bkj}$	Global benefit of increasing $U_{Bkj}$ towards the total objective function	(12)
$B_{PAkj}$	Benefit of increasing $U_{Akj}$ towards the power and ripple objective function	(8)
$B_{PBkj}$	Benefit of increasing $U_{Bkj}$ towards the power and ripple objective function	(9)
$B_{Vkj}$	Benefit of increasing $U_{kj}$ towards the voltage objective	(4)
$C_{kj}$	DC-Link capacity of the *k*-th phase *j*-th module	Figure 1
f	Global objective function	(10)
$f_{P}$	Objective function for the power regulation and ripple reduction alone	(7)
$F_{V}$	Function to measure the (pondered) quadratic DC-Link voltage deviation	(1)
$f_{V}$	Objective function for DC-Link voltage regulation alone	(3)
$G_{Pkj}$	Power and ripple gain for the *k*-th phase *j*-th module	(8) and (9)
$G_{Vkj}$	Voltage regulation gain for the *k*-th phase *j*-th module	(1)
$i_{\alpha }\;\&\;i_{\beta }$	Power-invariant α and β components of the phase currents ^2^ (^2^ The sign criteria from Figure 1 is selected for the voltages and currents there defined.)	
$i_{d}\;\&\;i_{q}$	Power-invariant d and q components of the phase currents ^2^ (^2^ The sign criteria from Figure 1 is selected for the voltages and currents there defined.)	
$i_{k}$	Current of the *k*-th phase ^2^ (^2^ The sign criteria from Figure 1 is selected for the voltages and currents there defined.)	Figure 1
j	Subscript ^1^ indicating the module inside the phase, j∈{1, 2, 3…N} (^1^ Subscripts other than j and k do not represent any numerical values.)	
k	Subscript ^1^ indicating the phase, k∈{1, 2, 3} (^1^ Subscripts other than j and k do not represent any numerical values.)	
L	Phase inductance	Figure 1
N	Number of modules on each phase	
$p_{k}^{*}$	Balance power reference for the *k*-th phase ^3^ (^3^ $p_{k}^{*}$ and $v_{0}$ belong to the method from [28] and are introduced on Section 3.2.2.)	
$P_{kj}$	Actual active power received by the *k*-th phase *j*-th module	
$P_{kj}{}^{*}$	Desired active power received by the *k*-th phase *j*-th module	
$U_{Akj}$	Positive output voltage deviation of the *k*-th phase *j*-th module from $U_{kj}{}^{*}$	(6)
$U_{Bkj}$	Negative output voltage deviation of the *k*-th phase *j*-th module from $U_{kj}{}^{*}$	(6)
$U_{kj}$	Voltage output selected for the *k*-th phase *j*-th module ^2^ (^2^ The sign criteria from Figure 1 is selected for the voltages and currents there defined.)	Figure 1
$U_{kj}{}^{*}$	Voltage output for the *k*-th phase *j*-th corresponding to its desired power	(5)
$U_{Tk}{}^{*}$	Total voltage reference for the *k*-th phase given by the current regulator	
$U_{Tk}{}^{’}$	$U_{Tk}{}^{*}$ after discounting the voltage corresponding to the desired power	(14)
$v_{0}$	Zero-sequence voltage ^3^ (^3^ $p_{k}^{*}$ and $v_{0}$ belong to the method from [28] and are introduced on Section 3.2.2.)	(17)
$V_{Gk}$	Voltage ^2^ of the grid *k*-th phase (^2^ The sign criteria from Figure 1 is selected for the voltages and currents there defined.)	Figure 1
$V_{kj}$	Actual DC-Link voltage of the *k*-th phase *j*-th module	Figure 1
$V_{kj}{}^{*}$	Desired DC-Link voltage of the *k*-th phase *j*-th module	

## References

[1] Franquelo L.G., Rodriguez J., Leon J.I., Kouro S., Portillo R., Prats M.A.M. (2008). The age of multilevel converters arrives. IEEE Ind. Electron. Mag..

[2] Yu Y., Konstantinou G., Hredzak B., Agelidis V.G. (2016). Power balance of cascaded H-bridge multilevel converters for large-scale photovoltaic integration. IEEE Trans. Power Electron..

[3] Xiong L., Gui Y., Liu H., Yang W., Gong J. A hybrid CHB multilevel inverter with supercapacitor energy storage for grid-connected photovoltaic systems. Proceedings of the 2018 IEEE Applied Power Electronics Conference and Exposition (APEC).

[4] Tafti H.D., Maswood A.I., Konstantinou G., Townsend C.D., Acuna P., Pou J. (2018). Flexible control of photovoltaic grid-connected cascaded H-bridge converters during unbalanced voltage sags. IEEE Trans. Ind. Electron..

[5] Yu Y., Konstantinou G., Townsend C., Aguilera R.P., Agelidis V.G. (2017). Delta-connected cascaded H-bridge multilevel converters for large-scale photovoltaic grid integration. IEEE Trans. Ind. Electron..

[6] Kumar T.S., Shanrnugam J. Design and anlaysis of CHB inverter by using Pi fuzzy and ANN techniques with grid connected PV System. Proceedings of the 2020 IEEE International Conference for Innovation in Technology (INOCON).

[7] Gomez-Merchan R., Vazquez S., Alcaide A.M., Tafti H.D., Leon J.I., Pou J., Rojas C.A., Kouro S., Franquelo L.G. (2021). Binary search based flexible power point tracking algorithm for photovoltaic systems. IEEE Trans. Ind. Electron..

[8] Romero-Cadaval E., Spagnuolo G., Franquelo L.G., Ramos-Paja C.A., Suntio T., Xiao W. (2013). Grid-connected photovoltaic generation plants: Components and operation. IEEE Ind. Electron. Mag..

[9] Nasiri M.R., Farhangi S., Rodriguez J. (2019). Model predictive control of a multilevel CHB STATCOM in wind farm application using diophantine equations. IEEE Trans. Ind. Electron..

[10] Rodriguez E., Farivar G.G., Beniwal N., Townsend C.D., Tafti H.D., Vazquez S., Pou J. (2020). Closed-loop analytic filtering scheme of capacitor voltage ripple in multilevel cascaded H-bridge converters. IEEE Trans. Power Electron..

[11] Ge X., Gao F. (2018). Flexible third harmonic voltage control of low capacitance cascaded H-bridge STATCOM. IEEE Trans. Power Electron..

[12] Neyshabouri Y., Chaudhary S.K., Teodorescu R., Sajadi R., Iman-Eini H. (2020). Improving the reactive current compensation capability of cascaded H-bridge based STATCOM under unbalanced grid voltage. IEEE J. Emerg. Sel. Top. Power Electron..

[13] Sajadi R., Iman-Eini H., Bakhshizadeh M.K., Neyshabouri Y., Farhangi S. (2018). Selective harmonic elimination technique with control of capacitive DC-link voltages in an asymmetric cascaded H-bridge inverter for STATCOM application. IEEE Trans. Ind. Electron..

[14] Marzoughi A., Neyshabouri Y., Imaneini H. (2014). Control scheme for cascaded H-bridge converter-based distribution network static compensator. IET Power Electron..

[15] Qiu W., Zheng Z., Guo M., Chen Z. An interconnection strategy for flexible arc-suppression device based on cascaded H-bridge converter in distribution network. Proceedings of the 2018 China International Conference on Electricity Distribution (CICED).

[16] Raveendran V., Andresen M., Buticchi G., Liserre M.G. (2020). Thermal stress based power routing of smart transformer with CHB and DAB converters. IEEE Trans. Power Electron..

[17] Marquez A., Leon J.I., Monopoli V.G., Vazquez S., Liserre M., Franquelo L.G. (2020). Generalized harmonic control for CHB converters with unbalanced cells operation. IEEE Trans. Ind. Electron..

[18] Alcaide A.M., Leon J.I., Portillo R., Yin J., Luo W., Vazquez S., Kouro S., Franquelo L.G. (2021). Variable-Angle PS-PWM Technique for multilevel cascaded H-bridge converters with large number of power cells. IEEE Trans. Ind. Electron..

[19] Leon J.I., Dominguez E., Wu L., Alcaide A.M., Reyes M., Liu J. (2021). Hybrid energy storage systems: Concepts, advantages, and applications. IEEE Ind. Electron. Mag..

[20] Monopoli V.G., Alcaide A.M., Leon J.I., Liserre M., Buticchi G., Franquelo L.G., Vazquez S. (2021). Applications and modulation methods for modular converters enabling unequal cell power sharing: Carrier variable-angle phase-displacement modulation methods. IEEE Ind. Electron. Mag..

[21] Liu Y., Huang A.Q., Song W., Bhattacharya S., Tan G. (2009). Small-signal model-based control strategy for balancing individual DC capacitor voltages in cascaded multilevel inverter-based STATCOM. IEEE Trans. Ind. Electron..

[22] Lizana R., Perez M.A., Arancibia D., Espinoza J.R., Rodriguez J. (2015). Decoupled current model and control of modular multilevel converters. IEEE Trans. Ind. Electron..

[23] Ayob S.M., Salam Z., Jusoh A. Trapezoidal PWM scheme for cascaded multilevel inverter. Proceedings of the IEEE International Power Energy Conference.

[24] Ye M., Ren W., Chen L., Wei Q., Song G., Li S. (2019). Research on power-balance control strategy of CHB multilevel inverter based on TPWM. IEEE Access.

[25] Sreenivasarao D., Agarwal P., Das B. (2014). Performance evaluation of carrier rotation strategy in level-shifted pulse-width modulation technique. IET Power Electron..

[26] Angulo M., Lezana P., Kouro S. Level-shifted PWM for cascaded multilevel inverters with even power distribution. Proceedings of the IEEE Power Electronics Specialists Confefeence (PESC).

[27] Hu Y., Zhang X., Mao W., Zhao T., Wang F., Dai Z. (2020). An optimized third harmonic injection method for reducing DC-link voltage fluctuation and alleviating power imbalance of three-phase cascaded H-bridge photovoltaic inverter. IEEE Trans. Ind. Electron..

[28] Aguilera R.P., Acuna P., Rojas C.A., Konstantinou G., Pou J. Instantaneous zero sequence voltage for grid energy balancing under unbalanced power generation. Proceedings of the 2019 IEEE Energy Conversion Congress and Exposition (ECCE).

[29] Townsend C.D., Summers T.J., Betz R.E. Control and modulation scheme for a Cascaded H-Bridge multi-level converter in large scale photovoltaic systems. Proceedings of the 2012 IEEE Energy Conversion Congress and Exposition (ECCE).

[30] Qi C., Chen X., Tu P., Wang P. (2018). Cell-by-cell-based finite-control-set model predictive control for a single-phase cascaded H-bridge rectifier. IEEE Trans. Power Electron..

[31] Galván L., Galván E., Carrasco J.M. Optimal modulation method for DC-link control in cascaded H-bridge multilevel converters. Proceedings of the IECON 2019—45th Annual Conference of the IEEE Industrial Electronics Society.

[32] Gómez P.J., Galván L., Galván E., Carrasco J.M. Energy storage systems current ripple reduction for DC-link balancing method in hybrid CHB topology. Proceedings of the IECON 2020 the 46th Annual Conference of the IEEE Industrial Electronics Society.

[33] Montero-Robina P., Marquez A., Dahidah M., Vazquez S., Leon J.I., Konstantinou G.G., Franquelo L.G. (2022). Feedforward modulation technique for more accurate operation of modular multilevel converters. IEEE Trans. Power Electron..

